# A Retrospective Study Assessing the Clinical Outcomes After Cheilectomy and Subchondroplasty for Hallux Rigidus

**DOI:** 10.7759/cureus.43446

**Published:** 2023-08-14

**Authors:** Don Koh, Darshana Chandrakumara, Raj Socklingam, Charles Kon Kam King

**Affiliations:** 1 Orthopaedics, Changi General Hospital, Singapore, SGP

**Keywords:** subchondroplasty, cheilectomy, primary osteoarthritis, foot & ankle surgery, hallux disorders, hallux rigidus

## Abstract

Introduction

Hallux rigidus (HR) refers to osteoarthritis of the first metatarsal phalangeal joint, resulting in stiffness, pain, and limitation in daily function. Surgery of HR is indicated in those who have failed a trial of non-operative management and is typically divided into joint-preserving (JP) and joint-sacrificing procedures. Cheilectomy is the most commonly practiced JP procedure, often done in conjunction with associated procedures for HR. Our paper aims to report the clinical outcomes after cheilectomy and cheilectomy done with subchondroplasty (SCP) performed for HR.

Methods

All patients who underwent cheilectomy for HR between 2017 and 2022 were identified and had their outcomes evaluated at the time of this review. The patients had their pre-operative radiographs and clinical and operative notes analyzed for the grading of HR. Functional outcomes were assessed with the use of the visual analog scale (VAS) and American Orthopaedic Foot and Ankle Society (AOFAS) scores, as well as comparing the pre-operative and post-operative degree of dorsiflexion of the affected first metatarsophalangeal joint. This study was approved by the SingHealth Institutional Review Board (IRB) Institution with approval number 2021/2629.

Results

A total of 19 patients and 20 feet were included in our study at a mean follow-up of 29.8 months. There was an increase in dorsiflexion of the first MTP joint by 27.2 degrees *(p-value = <0.0001)*. Patients who underwent cheilectomy alone (Group 1) had a mean improvement in VAS scores of 5.46 (*p-value = <0.0001*). Patients who underwent SCP of the first metatarsal head along with cheilectomy (Group 2) had an improvement in VAS scores by 5.78 (*p-value = 0.0007*). There was a mean improvement in AOFAS scores of 25.6 (*p-value = <0.0001*) for patients in Group 1. Patients in Group 2 had a mean improvement in AOFAS scores of 31.0 (*p-value = 0.0003*).

Conclusion

Both cheilectomy and cheilectomy performed with SCP for HR show good outcomes at short-term follow-up (mean 29.8 months). Cheilectomy is a viable alternative to arthrodesis for the surgical treatment of HR even in patients with higher grades. The use of SCP should be further explored as an adjunct in the surgical treatment of HR.

## Introduction

The first metatarsophalangeal (MTP) joint is the most common site of osteoarthritis (OA) in the foot and is referred to by the term hallux rigidus (HR) [1]. The degenerative nature of OA results in a reduced range of motion at the joint, pain, and functional limitation [2] and has a prevalence of nearly 10% [3], thus representing a huge burden.

As with OA affecting the other joints, a trial of non-operative management for HR is recommended as the first line of treatment [4]. These measures include the use of intra-articular injections, orthoses, and physiotherapy. In patients whose symptoms still persist, surgical management is then indicated. Surgical options for HR are generally categorized into joint sacrificing or joint sparing procedures. Joint-sacrificing surgery includes arthrodesis or arthroplasty, while joint-sparing surgery involves cheilectomy and phalanx or metatarsal osteotomies [5].

Cheilectomy involves the debridement of the dorsal one-third of the articular surface at the affected first MTP joint, as well as any surrounding osteophytes. Since it was first described in 1959, cheilectomy has been commonly practiced with good clinical outcomes [6]. Other associated procedures have been described for HR, the most notable of which is the Moberg osteotomy [7], which has shown good outcomes when performed in conjunction with a cheilectomy [8,9]. The dorsal closing wedge osteotomy acts to translate the MTP joint plantarward on the metatarsal head, to shift the arc of motion toward dorsiflexion at the expense of plantarflexion. The benefits of a Moberg osteotomy are thus improved dorsiflexion at the joint and offloading of areas with damaged cartilage without compromising joint contact area or biomechanics [10]. 

The purpose of our paper is therefore to explore the use of additional procedures which can be performed in conjunction with cheilectomy for the treatment of HR to further improve patient outcomes after surgery. In our study, we evaluate the use of subchondroplasty (SCP) in addition to cheilectomy.

## Materials and methods

All patients who underwent surgical management for HR between 2017 and 2022 at the author’s institution (Changi General Hospital, Singapore) were identified. Following the application of predetermined inclusion and exclusion criteria, patients who were included finally in this study had their pre-operative radiographs and their clinical and operative notes retrospectively analyzed. The patients were also invited back for a final review, to assess their functional outcomes pre-operatively and post-operatively with the use of the American Orthopaedic Foot and Ankle Society (AOFAS) and visual analog scale (VAS) scores.

All patients who underwent cheilectomy with or without additional procedures were included in this study. Other inclusion criteria included having had a pre-operative plain film radiograph performed, as well as having their clinical and operative notes accessible. Patients who had suffered new injuries to the same foot after the initial operation or had conditions affecting the contralateral foot resulting in functional impairment were excluded from this study. Patients who were unable to be contacted at the time of this review to complete final follow-up functional scoring were also excluded.

This study was approved by the SingHealth Institutional Review Board (IRB) Institution with approval number 2021/2629.

Coughlin and Shurnas classification

Pre-operative plain film radiographs of the operated foot of the patients were analyzed independently by the four authors and graded accordingly based on the classification system as described by Coughlin and Shurnas in 2003 [11] (Table 1). The examination findings from the patient's clinical notes were retrospectively analyzed for the grading of HR.

**Table 1:** Coughlin and Shurnas classification ROM: Range of motion

Grade	Examination findings	Radiographic findings
0	Stiffness	Normal
I	Mild pain at extremes of motion	Mild dorsal osteophyte, normal joint space
II	Moderate pain with ROM, increasingly more constant	Moderate dorsal osteophyte, <50% joint space narrowing
III	Significant stiffness, pain at extreme ROM, no pain at mid-range	Severe dorsal osteophyte, >50% joint space narrowing
IV	Significant stiffness, pain at extreme ROM, pain at mid-range of motion	Same as grade III

First MTP joint dorsiflexion

A passive range of motion at the affected first MTP joint was assessed using a goniometric technique [12]. The degree of dorsiflexion was taken by measuring the angle between the lines drawn by bisecting the proximal hallux and the first metatarsal in the sagittal plane after reaching the maximum range of dorsiflexion with a passive force applied by the examiner.

Functional scores

Functional outcomes were assessed through the use of the AOFAS and VAS scores. The VAS score was calculated based on a self-reported measure of the amount of pain that is being caused by a condition. The score is represented as a continuum, on a line ranging from one to ten, corresponding to the severity experienced by the patient. A score of one represents no pain, while a score of 10 represents the maximum amount of pain experienced possible [13].

The AOFAS score was calculated through subjective and objective assessment from both the patient and the examiner. The amount of pain experienced, as well as its function, is self-reported by the patient and is considered along with stability and alignment noted during physical examination, to give an overall score that represents the patient’s functional outcomes [14].

Data management and statistical analysis

Information collected during the course of our research was meticulously recorded and stored in offline servers at our institution as encrypted files to ensure data security.

An individual statistician conducted statistical analysis, using the GraphPad Prism (v.6.04) software. p-values were calculated and extracted using the paired sample t-test. The mean, standard deviation, and confidence intervals were calculated, and graphs were plotted to represent the data. 

The three measures of clinical outcomes in terms of first MTP joint dorsiflexion, AOFAS, and VAS scores are discussed individually in the paper. Results were considered significant if *p-values* are less than 0.05.

## Results

Patients

A total of 19 patients were included in this study, 9 males and 10 females. The average BMI of the patients was 26.2kg/m^2^ (range: 20.0 - 34.7 kg/m^2^), and the average age at the time of surgery was 57.1 (range: 35 - 73 years). A total of 20 feet were operated on, 15 right and 5 left. The etiology of HR was idiopathic in 18 and gout in 2. The mean time of follow-up was 29.8 months (range: 10.5 - 47.5). The aforementioned information is summarized in Table 2.

**Table 2:** Patient demographics

Measurement		Value
No. of patients		19
No. of feet		20
Age (Yr)		57.1 ± 11.6 (range: 35 – 73 years)
Sex	Male	9 (45.0%)
	Female	11 (55.0%)
Body mass Index (kg/m^2^)		26.2 ± 3.4 (range: 20.0 – 34.7 kg/m^2^)
Laterality	Right	15 (75.0%)
	Left	5 (25.0%)
Etiology	Idiopathic	18 (90.0%)
	Gout	2 (10.0%)
Time to follow up (months)		29.8 ± 9.6 (Range 10.5 – 47.5)

Surgical procedure

Patients were divided into two groups based on the surgical procedure they had undergone. Group 1 had 11 (of 20) feet, consisting of those who underwent a traditional cheilectomy alone for HR. Group 2 consisted of nine (of 20) feet, belonging to patients who had SCP performed in conjunction with a traditional cheilectomy. The information is displayed in Table 3.

**Table 3:** Patient grouping

Group	Surgical procedure	HR grade	Number of feet
1	Cheilectomy	2	8 (40.0%)
		3	3 (15.0%)
2	Cheilectomy + Subchondroplasty	2	4 (20.0%)
		3	5 (25.0%)

For the patients in Group 2, cheilectomy was first performed, followed by SCP. SCP was performed by first placing a cannula in the dorsal surface of the first metatarsal head under fluoroscopic guidance, followed by the application of the bone substitute material (BSM) into the defect.

Dorsiflexion

**Figure 1:**
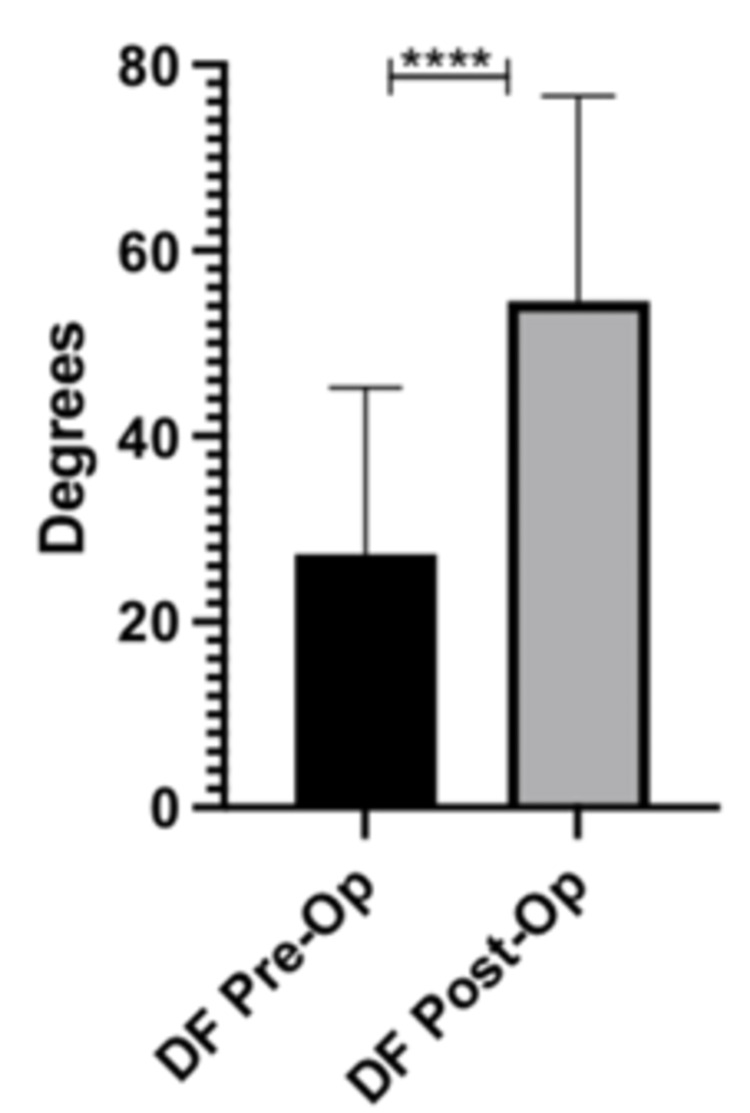
First MTP joint DF pre-op vs. post-op DF: Dorsiflexion; MTP: metatarsophalangeal

The average degree of dorsiflexion at the first MTP joint pre-operatively was 27.3 degrees. All 20 feet which underwent cheilectomy showed an increase in dorsiflexion of the first MTP joint by 27.2 degrees (p-value = <0.0001). The information is displayed in Figure 1 and Table 4.

**Table 4:** First MTP joint dorsiflexion DF: Dorsiflexion; SD: standard deviation; CI: confidence interval; MTP: metatarsophalangeal

DF (degrees)	Values (degrees, SD)	p-value
Pre-Op (degrees)	27.3 ± 17.1 SD	-
Post-Op (degrees)	54.6 ± 21.1 SD	-
Change in DF (degrees)	27.2 ± 13.5 (95% CI 18.2 – 36.3)	<0.0001

VAS scores

**Figure 2:**
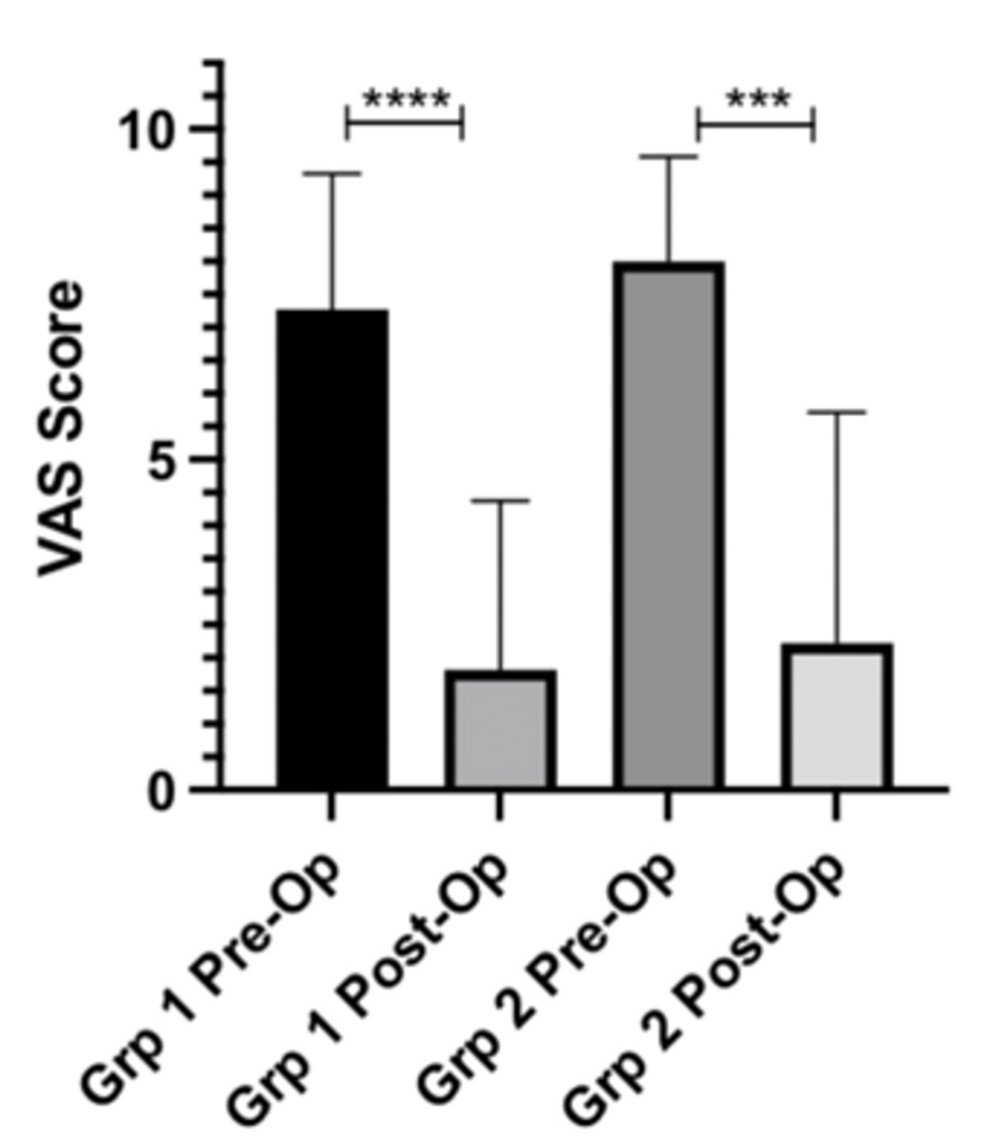
VAS scores pre-op vs. post-op VAS: Visual analog scale; Grp 1: cheilectomy; Grp 2: cheilectomy and Subchondroplasty

Patients in Group 1 (cheilectomy) had a mean improvement in VAS scores by 5.46 (p-value = <0.0001). Patients in Group 2 (cheilectomy and SCP) had a mean improvement in VAS scores by 5.78 (p-value = 0.0007). The above information is displayed in Figure 2 and Table 5.

**Table 5:** VAS scores pre-op vs. post-op VAS: Visual analog scale; Group 1: cheilectomy; Group 2: cheilectomy and subchondroplasty

VAS score	Group	Values (number, SD (CI)	p value
Pre-op	Group 1	7.27 ± 1.96 SD	-
	Group 2	8.00 ± 1.49 SD	-
Post-op	Group 1	1.82 ± 2.44 SD	-
	Group 2	2.22 ± 3.29 SD	-
Change in VAS Score	Group 1	5.46 ± 2.58 (95% CI 3.72 – 7.19)	<0.0001
	Group 2	5.78 ± 3.27 (95% CI 3.26 – 8.29)	0.0007

AOFAS scores

**Figure 3:**
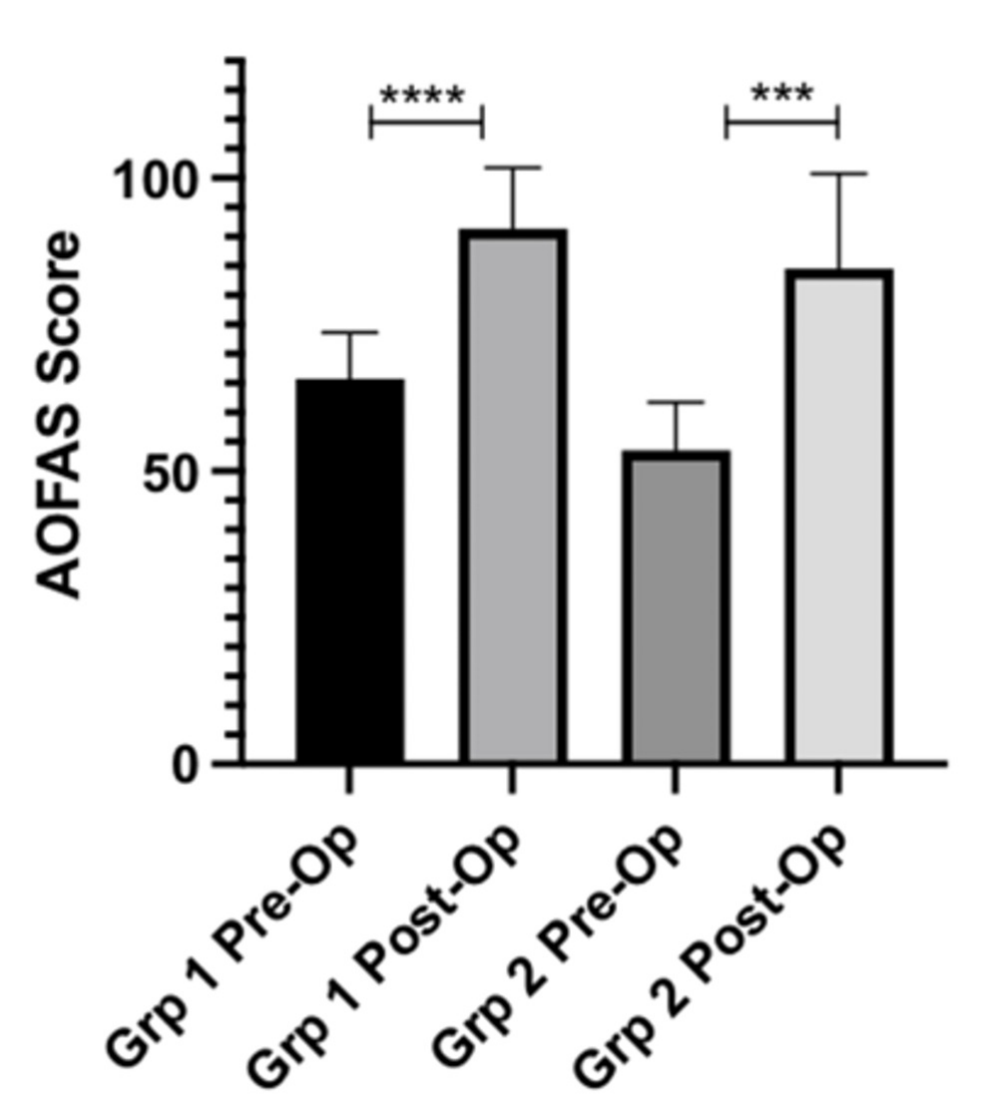
AOFAS scores pre-op vs. post-op AOFAS: American Orthopaedic Foot and Ankle Society; Grp 1: cheilectomy; Grp 2: cheilectomy and subchondroplasty

There was a mean improvement in AOFAS scores of 25.6 (p-value = <0.0001) for patients in Group 1 (cheilectomy alone). Patients in Group 2 (cheilectomy and SCP) had a mean improvement of 31.0 (p-value = 0.0003). Figure 3 and Table 6 display the information described above.

**Table 6:** AOFAS scores pre-op vs. post-op AOFAS: American Orthopaedic Foot and Ankle Society; Group 1: cheilectomy; Group 2: cheilectomy and subchondroplasty

AOFAS scores	Group	Values, SD (CI)	p value
Pre-op	Group 1	65.7 ± 7.56 SD	
	Group 2	53.6 ± 7.76 SD	
Post-op	Group 1	91.4 ± 9.87 SD	
	Group 2	84.6 ± 15.3 SD	
Change in AOFAS Score	Group 1	25.6 ± 9.86 (95% CI 19.0 – 32.3)	<0.0001
	Group 2	31.0 ± 15.2 (95% CI 19.3 – 42.7)	0.0003

## Discussion

Good outcomes at short- and medium-term follow-ups were seen in our patients after cheilectomy for HR, consistent with current literature. Cheilectomy has been shown in multiple studies to have consistently good outcomes in those with lower grades of HR [11]. For higher grades of HR, however, treatment still varies with some preferring joint-sacrificing procedures, namely, arthrodesis, over cheilectomy [5]. Our study reports good improvement in VAS and AOFAS scores even for patients with higher grade HR (Grade 3), adding to the growing evidence supporting the use of cheilectomy as a viable option for even those with more advanced disease [15].

The joint-sparing nature of cheilectomy allows room, in the event of poor outcomes, for revision surgery or conversion to joint-sacrificing arthrodesis or arthroplasty. In addition, cheilectomy avoids the possible complications of arthrodesis such as the risk of non-union and implant prominence causing pain [16]. Lastly, many patients are reluctant to undergo fusion due to having to lose movement at the joint. Therefore, there is a role for cheilectomy to be explored as a first-line surgical option for HR.

Repetitive stress of a joint leads to the formation of a bone marrow lesion (BML) through the remodeling of the subchondral bone, resulting in an area of reduced mineralization, along with increased necrosis and fibrosis [17,18]. The severity of BMLs of the metatarsal head and proximal phalanx was shown to be associated with the presence of HR based on magnetic resonance imaging (MRI) [19], and its presence has been strongly correlated with increased severity of symptoms in patients with OA [20].

SCP is a relatively new procedure, which involves the injection of the BSM into these symptomatic BMLs to improve structural integrity, stimulate healing, and reduce pain [21]. The use of SCP in other joints commonly affected by OA, most notably the knee, has seen good outcomes in the form of reduced pain, improved function, and delay in time till conversion to arthroplasty, as well as reduced sizes of the BMLs on follow-up MRI [22,23].

Data regarding the use of SCP in the first MTP joint are drastically lacking, with only a few studies having described the use of SCP in the foot and ankle [24]. The use of SCP alone in the first metatarsal head was shown to have unfavorable outcomes in a study by Sharma et al., who reported the development of the known complication of symptomatic avascular necrosis in two of their patients [23,25].

Our study shows good outcomes following both cheilectomy alone and cheilectomy done in conjunction with SCP at short- and medium-term follow-ups. No complications were seen in our patients after SCP, in contrast to what was previously described when used in the first MTP joint. SCP should be further explored for use in the treatment of HR as the early results are promising.

Limitations

Our paper is not without limitations. Firstly, all surgeries were performed by a single surgeon in the author’s institution. Secondly, our paper is a retrospective study and not blinded, leading to multiple biases. Thirdly, the sample size is small, with follow-up only in short and medium term.

The patient population can be followed up in the long term to evaluate for outcomes after cheilectomy and SCP, as well as assess for any late complications.

Additionally, the study can be replicated in a prospective single-blind study in a large sample size to evaluate the differences in outcome in SCP is performed in addition to a traditional cheilectomy, and whether it has better outcomes compared to other associated procedures such as a Moberg osteotomy. 

## Conclusions

Both cheilectomy and cheilectomy performed with SCP for HR shows good outcomes at short-term follow-up (mean 29.8 months). Cheilectomy is a viable alternative to arthrodesis for the surgical treatment of HR even in patients with higher grades. The use of SCP should be further explored as an adjunct in the surgical treatment of HR.
